# Genome-Wide Association and Transcriptome-Wide Association Studies Identify Novel Susceptibility Genes Contributing to Colorectal Cancer

**DOI:** 10.1155/2022/5794055

**Published:** 2022-07-01

**Authors:** Ruimin Yin, Binbin Song, Jingjing Wang, Chaodan Shao, Yufen Xu, HongGang Jiang

**Affiliations:** ^1^Department of General Surgery, Zhejiang Hospital, 12# Lingyin Road, Zhejiang, 310013 Hangzhou, China; ^2^Department of Oncology, The Affiliated Hospital of Jiaxing University, 1882# Zhonghuan South Road, Jiaxing 314000, China; ^3^Department of Gastrointestinal Surgery, The Affiliated Hospital of Jiaxing University, 1882# Zhonghuan South Road, Jiaxing 314000, China

## Abstract

**Background:**

Colorectal cancer (CRC) is among the most common cancers diagnosed worldwide. Although genome-wide association studies have effectively identified the genetic basis of CRC, there is still unexplained variability in genetic risk. Transcriptome-wide association studies (TWAS) integrate summary statistics from CRC genome-wide association studies (GWAS) with gene expression data to prioritize these GWAS findings and uncover additional gene-trait correlations.

**Methods:**

First, we carried out a post-GWAS analysis using summary statistics from a large-scale GWAS of CRC (*n* = 4,562 cases, *n* = 382,756 controls). Second, combined with the expression weight sets from GTEx (v7), susceptibility genes were identified with the FUSION software. Colocalization, conditional and fine-mapping analyses, phenome-wide association study (pheWAS), and Mendelian randomization were employed to further characterize the observed correlations.

**Results:**

In the post-GWAS analyses, we first identified new genome-wide significant associations: three genomic risk loci were identified at 8q24.21 (rs6983267, *P* = 6.98 × 10^−12^), 15q13.3 (rs58658771, *P* = 1.40 × 10^−10^), and 18q21.1 (rs6507874, *P* = 1.91 × 10^−14^). In addition, the TWAS also identified four loci statistically significantly associated with CRC risk, largely explained by expression regulation, including six candidate genes (*DUSP10*, *POU5F1B*, *C11orf53*, *COLCA1*, *COLCA2*, and *GREM1-AS1*). We further discovered evidence that low expression of *COLCA2* is correlated with CRC risk with Mendelian randomization.

**Conclusions:**

We discovered novel CRC risk loci and candidate functional genes by merging gene expression and GWAS summary data, offering new insight into the molecular processes underlying CRC development. This makes it easier to prioritize potential genes for follow-up functional research in CRC.

## 1. Introduction

Colorectal cancer (CRC) is one of the most common cancers identified globally [1], accounting for around 10% of all cancers and cancer-related deaths identified each year, with over 1.2 million people detected with CRC each year and millions of deaths from CRC each year [2]. Notably, in some high-income countries, such as Australia, Canada, the United States, and some European countries, 5-year survival rates have reached nearly 65%, but in low-income countries, 5-year relative survival rates are still less than 50%, and survival rates decline with age [3–5].

Unlike monogenic diseases, CRC is a polygenic disease produced by genetic inheritance and environmental factors (such as obesity, physical inactivity, poor diets, alcohol consumption, and smoking) which play a major part in the etiology of both familial and sporadic CRC [6–8]. Twin family studies have shown heritability of about 33%-48% for CRC, implying that a major genetic component is generating the phenotypic variation [9]. Patients with CRC who have a positive family history represent approximately 10-20% of the total. The risk of disease varies with the quantity of relatives of patients with CRC, the severity of the disease, and the age at which CRC is diagnosed [10, 11].

Several GWASs on CRC have discovered around 60 correlation signals at more than 50 loci over the past decades, while an increasing amount of single nucleotide polymorphisms (SNPs) have shown statistical significance but often only a little fraction of the risk for CRC risk [12–14]. Even though GWAS has been quite successful in identifying elements that contribute to the genetic architecture of CRC, the loci detected are generally difficult to characterize biologically. In contrast, a transcriptome-wide association study (TWAS) provides more interpretative biologically relevant findings owing to the usage of disease-relevant cell types and tissues, and also databases detailing tissue-specific expression [15]. TWAS could detect genes whose gene-regulated expression may be related to the risk of diseases by merging expression quantitative loci (eQTL) results with GWAS summary data [15].

To identify genetically regulated risk loci associated with CRC, we performed gene-based and gene-set tests utilizing the CRC GWAS summary statistics from the publicly available UK Biobank with the FUMA online tool, an available online website at http://fuma.ctglab.nl [16, 17]. Then, we also leverage the currently available CRC GWAS summary statistical to conduct a TWAS; the cohort includes 4,562 CRC cases and 382,756 controls from Europe. Possibly relevant tissue-derived transcriptomic expression weights were employed, comprising the whole blood and 2 CRC-relevant tissue (colon transverse and colon sigmoid) panels from GTEx (v7). Subsequently, we conducted the conditional analysis of all significant TWAS correlations to identify the jointly significant TWAS genes (i.e., the driven genes at each risk locus). Follow-up analyses, including summary data-based Mendelian randomization (SMR), colocalization analysis, fine-mapping analysis, and phenome-wide association study (pheWAS), were performed to broadly characterize the associations identified. Altogether, our findings demonstrate TWAS's ability to identify CRC risk genes with small effect sizes and present a testable target for future functional validation of CRC.

## 2. Method

### 2.1. Study Cohort

Included in this analysis were the following data: (i) genome-wide summary data from the GWAS of CRC by Zhou et al. [17], (ii) 3 SNP weight sets from GTEx (v7) transcriptomic reference samples, and (iii) the 1000 Genomes Project reference for linkage disequilibrium (LD) estimation.

First, we utilized the CRC GWAS summary data from UK Biobank analysis results (https://www.leelabsg.org/resources), in which individuals (*n* = 4,562 cases, *n* = 382,756 controls) had European genetic heritage [17]; second, SNP weight sets from the relevant tissues were used. SNP weight sets indicate the correlation of the SNP with its annotated gene expression [15]; SNP weight sets from colon transverse, colon sigmoid, and whole blood were obtained from the FUSION website (http://gusevlab.org/projects/fusion/); third, the LD reference of the1000 Genomes Phase 3 European (*N* = 489) was also obtained from the FUSION website (http://gusevlab.org/projects/fusion/).

### 2.2. Identification of Independent SNPs and Lead SNPs with FUMA

Utilizing information from multiple biological resources, FUMA has established a range of SNP functional annotation methods on the website platform, providing functional annotations of GWAS association signals, gene prioritization [16]. The identification of lead SNPs and candidate SNPs is based on the following criteria: (i) independently significant SNPs were identified by *P* < 5 × 10^−8^ and independent from each other at *r*^2^ < 0.6; (ii) independent lead SNPs were identified as independent significant SNPs and independent from each other at *r*^2^ < 0.1; (iii) genomic risk loci was identified by merging lead SNPs within a 250 kb window and all known SNPs in LD of *r*^2^ ≥ 0.6 with one of the independent significant SNPs; and (iv) the1000 Genomes Phase 3 European was defined as the reference panel population [16].

### 2.3. MAGMA for Gene-Based and Gene-Set Tests

Gene-based tests/gene-set analyses are methods capable of summarizing SNP associations at the gene level and associating gene sets with biological pathways. To determine prospective candidate genes and biological processes enriched for markers with low but not necessarily genome-wide significant *P* values in GWAS, gene-based tests/gene-set analyses were conducted by MAGMA, which was implemented in FUMA [16].

The CRC GWAS summary statistics were utilized in MAGMA's gene-based analysis to aggregate the association of SNPs within gene regions without accounting for SNP effects on gene expression [18]. The SNP-based *P* values from the CRC GWAS summary statistics were utilized as the input file for the gene-based analysis. For gene-based studies in MAGMA, we employed all 19,427 protein-coding genes from the NCBI 37.3 gene definition as the foundation (https://ctg.cncr.nl/software/magma). After SNP annotation, 19,252 genes had at least one SNP covering. Note that the LD relationship between SNPs was taken into account when performing the gene association test. We employed a strict Bonferroni correction to account for numerous testing, and the genome-wide threshold for significance was set at *P* = 2.60 × 10^−6^ (0.05/19252).

Gene-set tests were carried out in MAGMA utilizing competitive genomic analysis, which integrated the test statistics for all genes in a given genome to provide a joint association statistic, which was then implemented in FUMA [16]. This statistic was compared to that of all other genes not included in that set, while taking into consideration the quantity of SNPs within each gene, gene density, and differential sample size (unequal sample size contributing to each gene). 10,678 gene sets (curated gene sets: 4761, GO terms: 5917) from MsigDB v6.2, as well as a default competitive test model, were employed in the MAGMA gene-set analysis. Bonferroni correction was performed by employing a false discovery rate threshold of 5%.

### 2.4. Transcriptome-Wide Association Study

We used TWAS analyses with FUSION (http://gusevlab.org/projects/fusion) with default settings to identify genes whose gene-regulated expression may be related to the risk of CRC [15]. TWAS was carried out using reference panels obtained from tissue-specific gene expression and the CRC GWAS summary data [15], taking into account the LD structure between SNPs. To interpret the LD structure, we used the 1000 Genomes Phase 3 (European, *N* = 489) data as the LD reference panel. FUSION software was used to generate SNP weight sets from BLUP, BSLMM, LASSO, Elastic Net, and top SNPs utilizing genotype and expression data unless BLUP/BSLMM was eliminated owing to sample size or convergence issues [15]. A strict Bonferroni-corrected threshold was utilized: *P* < 1.11 × 10^−6^ (0.05/44,953) (considering the relations between features within and across SNP weight sets).

### 2.5. Bayesian Colocalization

To determine whether GWAS SNPs colocalized with eQTLs, Bayesian colocalization was examined using the COLOC package (https://cran.r-project.org/web/packages/coloc/, version 5.1.0) in R implemented by FUSION for all associations with *P*_TWAS_ < 0.05 within a 1 megabase (Mb) window [19]. This Bayesian colocalization technique revealed the posterior probability (PP) that relationships within a locus for two outcomes (GWAS and eQTL signals) were caused by a common causal variable or variants in strong LD. COLOC evaluated five hypotheses: PP0, no eQTL and GWAS association; PP1, association with eQTL, but no GWAS; PP2, association with GWAS, but no eQTL; PP3, eQTL and GWAS association, but independent signals; and PP4, shared eQTL and GWAS associations. The main objective is to determine whether the GWAS and eQTL signals are consistent with common causal variants (i.e., PP4). In reality, a high PP (PP4 > 80%) implies that the GWAS and eQTL signals colocalize [19].

### 2.6. Joint/Conditional Analysis and Permutation

To determine multiple correlated features within a locus (or the same feature from multiple tissues), we performed a conditional analysis and hopefully identified which were conditionally independent. Moreover, we also wondered how much the GWAS signal remained after the correlation of the function was excluded [20, 21]. This process identified which features indicate independent associations (called jointly significant) and which features were not significant when the predicted expression of the jointly significant genes in the region was ignored (called marginally significant) [20]. We also computed the extent to which GWAS correlations within each locus can be described by the functional connections detected in this TWAS. Additionally, the conditional analysis enables us to evaluate the extent to which the GWAS association signal within each locus may be described by the functional connections identified in this TWAS. To account for correlations between features within and across SNP weight sets, we randomized the eQTL weights and recalculated empirical association statistics conditional on GWAS effects by FUSION. In this study, 1,000 permutation tests were performed for each TWAS gene, setting the significance of the permutation test at *P* < 0.05 [15, 20].

### 2.7. TWAS Fine Mapping

Fine-mapping of CaUsal gene Sets (FOCUS) is software for fine-mapping transcriptome-wide correlation study statistics to genomic risk areas discovered by FUSION and producing a set of reasonable features interpreting the observed genomic risk. The software accepts GWAS summary data and eQTL weights as input, as well as FUSION findings and weights. FOCUS calculates the posterior inclusion probability (PIP) for each feature in the region of interest and decides whether TWAS-significant genes are included in the default 90% credible set, which is the set of features most likely to include causal features [22]. PIP values greater than 0.05 for each feature in the related region suggest that the feature is more likely to be causal than any other feature in the associated region [21]. Consistent with the TWAS analysis performed by the FUSION software, the FOCUS software used the same TWAS reference panel from FUSION.

### 2.8. Summary Data-Based Mendelian Randomization of CRC

We applied summary data-based Mendelian randomization (SMR) based on pooled data (https://cnsgenomics.com/software/smr/) to determine genetic signals correlated with phenotypic and gene expression variation, utilizing pooling from independent GWASs and eQTL weight data (colon sigmoid, colon transverse, whole blood from GTEx (v7), and Westra, CAGE eQTL summary data) that can be employed to evaluate whether the effects of genetic variation are mediated by gene expression levels [23]. This method employs the concept of Mendelian randomization (MR), a methodology for detecting causal effects [24]. The first step in the SMR approach is MR analysis, in which genetic variants (such as SNPs) were defined as instrumental variables, gene expression levels were defined as exposures, and traits were defined as outcomes [25]. To control the genome-wide type I error rate, *P*_SMR_ values were Bonferroni corrected for the number of genes tested, meaning that genes less than 1 Mb away from the GWAS lead SNPs were considered significant using the Bonferroni-corrected SMR significance threshold [26]. Following the SMR test, the heterogeneity independent instrument (HEIDI) test was used to determine whether the connection was attributed to a common causal variant rather than widespread LD across the genome [25]. Considering that this analysis is conservative for gene analysis and maintains fewer genes than when correcting for multiple testing, we did not correct for multiple testing and a *P*_HEIDI_ threshold of 0.05 for the HEIDI test was identified [23].

### 2.9. Phenome-Wide Association Studies

To determine phenotypes associated with the best eQTL in the locus identified via TWAS, a pheWAS was performed using publicly available data from the GWAS Atlas (https://atlas.ctglab.nl). Only the top phenotypes were recorded (excluding CRC). Accounting for the current GWAS Atlas website which contains a total of 3,302 unique traits, a Bonferroni-corrected cut-off of 1.68 × 10^−5^ (0.05/the number of unique traits) was used.

## 3. Result

### 3.1. Study Overview

**Figure 1:**
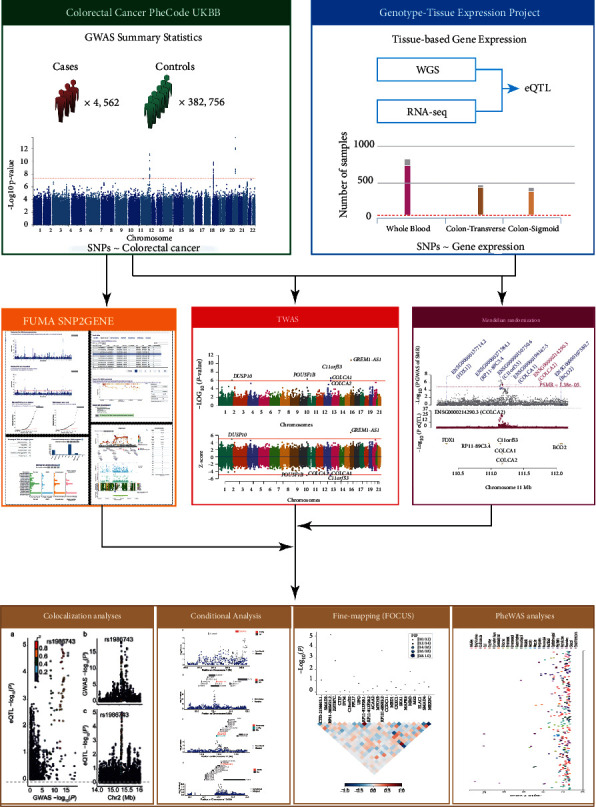
Schematic workflow of this study. We performed a TWAS for the CRC based on the publicly available GWAS datasets and the eQTL datasets. The GWAS datasets were derived from CRC GWAS summary statistics from UK Biobank analysis results; participants (*n* = 4,562 cases, *n* = 382,756 controls) were of European genetic ancestry. The eQTL dataset was from GTEx (v7). Follow-up analyses, including the SMR, colocalization analyses, conditional analysis, fine-mapping analysis, and pheWAS, were performed to extensively characterize the identified associations. CRC: colorectal cancer; eQTL: expression quantitative trait loci; GTEx: Genotype-Tissue Expression Project; GWAS: genome-wide association study; pheWAS: phenome-wide association study; SMR: summary data-based Mendelian randomization; SNP: single nucleotide polymorphism; UKBB: UK Biobank; WGS: whole-genome sequencing.

First, we used the FUMA online website and performed the post-GWAS analysis with CRC GWAS summary statistics as input files to identify independent significant SNPs and lead SNPs. Second, gene-based and gene-set tests were conducted using MAGMA and implemented in FUMA, combining the test statistics for all genes in a particular genome to obtain a joint association statistic [16]. After that, we used FUSION software to perform TWAS analysis to determine genes whose gene regulatory expression may be related to the risk of CRC with default settings [15]. Afterward, we conducted a Bayesian colocalization analysis for all associations with *P*_TWAS_ < 0.05 utilizing the COLOC package in R (https://cran.r-project.org/web/packages/coloc) implemented in FUSION to predict the PP association that an SNP contributed to the association signal in the GWAS and the eQTL [19]. Joint analysis was employed in regions with multiple significant associations to determine conditionally independent associations [22]. Beyond that, most genes remained significant following the permutation, indicating that their signal was genuine, not accidental. Finally, we used SMR, pheWAS, and FOCUS software to analyze and verify the above results [23] (Figure 1).

### 3.2. FUMA's Functional Annotation Analysis Results

To determine genetic loci that contribute to CRC and outcome, SAIGE, scalable and accurate software for generalized mixed-model association testing, was utilized to efficiently analyze a CRC case-control cohort, including a sample of 387,318 Caucasians of British European ancestry, with an imbalanced control case-control ratios and sample correlations [17]. A Manhattan plot of the GWAS results was drawn with the 28,146,008 SNPs that satisfied quality controls (Figure S1a). The quantile-quantile plot revealed an excellent match between the observed *P* value distributions and the predicted *P* value distribution by chance (*λ* = 1.035; Figure S1b), indicating that the genome-wide statistical results were not overinflated.

In the post-GWAS process, we first utilized the FUMA online website to conduct the functional annotation with the CRC GWAS summary statistics including 4,562 cases and 382,756 controls. After functional annotation analyses, we annotated 131 candidate SNPs that passed the gene-wide significance threshold (*P* < 5.00 × 10^−8^), and three independent lead SNPs were identified located at three genomic risk loci. These 131 independently significant SNPs were found in introns (32.3%, *P*_enrichment_ = 0.36) and noncoding RNA intronic (31.5%, *P*_enrichment_ = 9.39 × 10^−10^), intergenic (15.4%, *P*_enrichment_ = 7.68 × 10^−14^), and UTR3 (15.4%, *P*_enrichment_ = 1.54 × 10^−18^) regions that showed enrichment, while only 2.31% of the 131 independently significant SNPs were located in exonic regions and noncoding RNA exonic regions (Table S1, Figure S2).

One of the interesting genomic risk loci is 8q24.21, which contained 42 GWAS SNPs, representing 6 unique genes (*LINC01245*, *CCAT1*, *CASC21*, *CASC8*, *CCAT2*, and *POU5F1B*) (Figure S3a). As previously reported, SNPs in this region have been proven to be significantly correlated with CRC [27–29]. Another risk locus at 15q13.3 contained 5 unique protein-coding genes (*GOLGA8N*, *ARHGAP11A*, *SCG5*, *GREM1*, and *FMN1*) and 52 GWAS SNPs (Figure S3b). Consistent with the previous study, SNPs near *GREM1* and *FMN1* were highly correlated with elevated CRC risk [30], while the difference is that the rs4779584 reported is strongly related to an elevated risk of CRC, but the opposite in the present study (*P* = 1.73 × 10^−9^, OR = 0.85) [30]. In addition to the above two risk loci, there is also another genomic risk loci at 18q21.1. *CTIF*, *SMAD7*, and *DYM*, 3 protein-coding genes, were included in this locus. rs6507874 is the lead SNP of the genomic risk locus and is found in the intron of the *SMAD7* gene (Figure S3c), while the previous research that studied the relationship between rs6507874 and CRC risk showed that rs6507874 did not show a statistically significant connection (*P* = 0.075) with increased *SMAD7* expression [31].

### 3.3. Gene-Based and Gene-Set Tests Implemented in MAGMA

All SNPs found inside genes were assigned to 19,252 protein-coding genes in the gene-based analysis conducted with MAGMA. After gene-based analysis, 19252 genes had at least one SNP covering. The gene-based analysis for CRC summary statistics identified 5 genes (*SMAD7*, *COLCA1*, *COLCA2*, *POU5F1B*, and *LAMA5*) at a stringent Bonferroni correction for significance at *P* < 2.60 × 10^−6^ (Figure S4, Table S2). Immediately after, the results of the gene-set analysis conducted by MAGMA showed nonsignificant results after Bonferroni correction for numerous testing (Table S3). But it is worth noting that several suggested gene sets, such as neuroendocrine cell differentiation, linoleic acid (LA) metabolism, are known CRC-related pathways [32, 33].

### 3.4. Transcriptome-Wide Association Study

**Figure 2:**
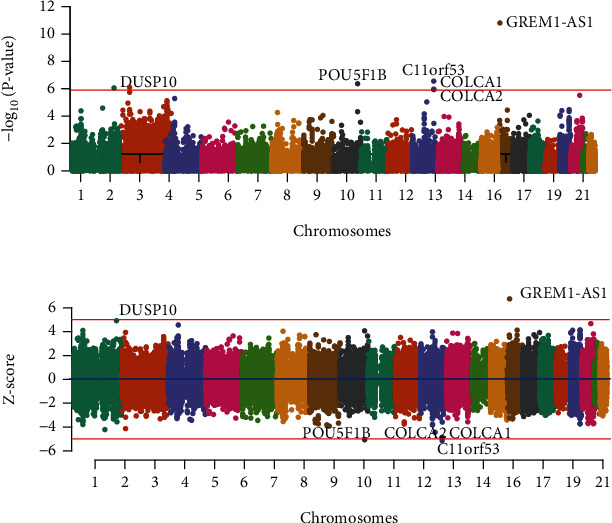
Manhattan plots of the TWAS results. (a) Gene-level Manhattan plot of the TWAS results from the European colorectal cancer cohort. The *x*-axis represents the genomic position (based on NCBI Build 37), and the *y*-axis shows the -log_10_ (*P* value). The red line represents the transcriptome-wide significance threshold (*P* = 1.11 × 10^−6^). (b) *Z*-scores of the TWAS results from the European colorectal cancer cohort. The *x*-axis represents the genomic position (based on NCBI Build 37), and the *y*-axis shows the *Z*-score from the association tests. The blue line indicates that *Z*-score is equal to 0. Red lines denote the Bonferroni-corrected significance threshold (∣*Z* | = 4.87, *P*_TWAS_ < 1.11 × 10^−6^). TWAS: transcriptome-wide association study.

To identify potential genes associated with CRC risk, we collected a publicly available GWAS dataset from a European case-control cohort. Of the 3 SNP weight sets (colon sigmoid, colon transverse, and whole blood), we identified 6 transcriptome-wide significant features (6 unique genes), with the colon sigmoid transcript-level weights yielding the most significant relationships (Table 1). Four transcriptome-wide significant loci were detected for 6 distinct genes (Figure 2).

### 3.5. Chromosome 1q41

**Figure 3:**
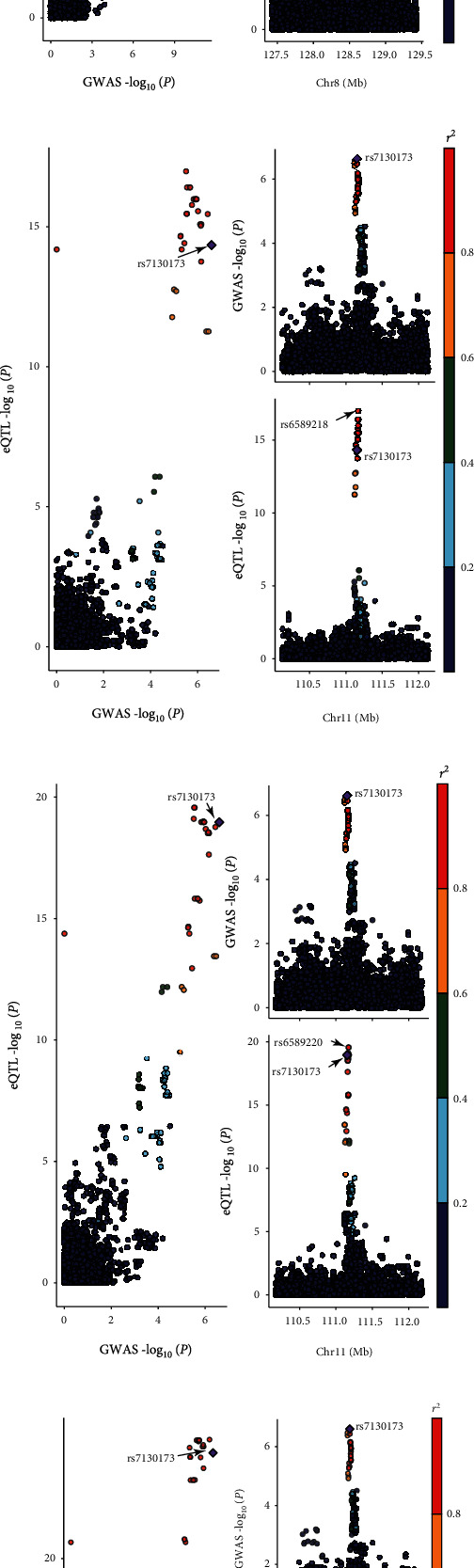
The locus-compare scatter plot for the association signals at *DUSP10*, *POU5F1B*, *C11orf53*, *COLCA1*, *COLCA2*, and *GREM1-AS1* in the European cohort. Colocalization analyses results are shown for (a) *DUSP10*, (b) *POU5F1B*, (c) *C11orf53*, (d) *COLCA1*, (e) *COLCA2*, and (f) *GREM1-AS1* genes. The locus-compare scatter plot compares the expression quantitative trait loci (eQTL) results and the genome-wide association study (GWAS) results, which indicates whether the GWAS top locus is also the leading SNP in the eQTL result. The eQTL results were from Genotype-Tissue Expression (GTEx) (v7). The GWAS results were from the European cohort (*n* = 4,562 cases, *n* = 382,756 controls). The gene prioritized in each locus is shown on the *y*-axis of the corresponding figure label. Chr.: chromosome.

Only one relevant feature in the locus, correlating to the DUSP10 gene, was shown to be transcriptome-wide significant (*P*_TWAS_ = 8.72 × 10^−7^) (Table 1). rs12125368, an intergenic variant, was the SNP in the locus most highly correlated with CRC (odds ratio (OR) = 1.34, *P*_GWAS_ = 2.65 × 10^−6^). And the best eQTL in the locus correlated with the expression level of the *DUSP10* gene (*P*_eQTL_ = 3.86 × 10^−4^) was rs6695584, which was in moderate LD with rs12125368 (0.6 ≥ *r*^2^ ≥ 0.4) (Figure 3(a)). Then, formal Bayesian colocalization indicated a moderate shared signal with a PP4 of 0.52 (Table 1), confirming the general likelihood that the GWAS and colon sigmoid eQTL signals may share the same variants at this locus.

To determine whether the signals of this locus were independent, we conducted conditional and joint analyses. The result showed that the *DUSP10* gene describes all of the signals at its loci (best SNP: rs12125368, *P*_GWAS_ = 2.65 × 10^−6^; conditioned on *DUSP10*, *P*_GWAS_ = 0.89) (Figure 3(a) and Figure S5a). FOCUS was utilized to assign a PIP to genes at each transcriptome-wide significant loci with relevant tissue in order to select putatively causal genes. Unfortunately, the PIP of *DUSP10* was not available in the three eQTL tissues from GTEx (v7) for this genomic locus 1q41 (Table 1).

### 3.6. Chromosome 8q24.21

*POU5F1B* (colon transverse) achieved transcriptome-wide significance, located at 8q24.21 locus (*P*_TWAS_ = 4.32 × 10^−7^). rs6983267, a noncoding transcript variant in the *CCAT2* gene or intron variant in the *CASC8* gene, was the most significant SNP associated with CRC at this locus (OR = 0.71, *P*_GWAS_ = 6.98 × 10^−12^). At this locus, rs7014346 was the best eQTL correlated with the expression level of the *POU5F1B* gene in the colon transverse tissue from the GTEx database (v7) (*P*_eQTL_ = 6.65 × 10^−7^), which was in moderate LD with rs6983267 (0.6 ≥ *r*^2^ ≥ 0.4) (Figure 3(b)). Colocalization analysis supported the fifth hypothesis with a PP4 of 0.73, offering suggestive evidence that the significant CRC GWAS signal and the expression level of the *POU5F1B* gene were driven by the same causal variant.

For the genomic locus 8q24.21, *POU5F1B* was added in the 90%-credible gene set with a PIP of 1 in the colon transverse (Table 1). At this locus, conditional and joint analyses found that *POU5F1B* explains 0.53 of the variance at its loci (best SNP: rs6983267, *P*_GWAS_ = 6.98 × 10^−12^; conditioned on *POU5F1B*, *P*_GWAS_ = 2.30 × 10^−6^) (Figure 3(b) and Figure S5b).

### 3.7. Chromosome 11q23.1

Three transcriptome-wide significant genes (*C11orf53*, *COLCA1*, and *COLCA2*) were observed within the q23.1 region of chromosome 11 (*P*_TWAS_ = 2.80 × 10^−7^, 1.08 × 10^−6^, and 1.11 × 10^−6^, respectively). rs7130173 is an intron variant, most significantly associated with CRC at its loci (OR = 0.76, *P*_GWAS_ = 2.52 × 10^−7^). Meanwhile, our study, respectively, identified rs6589218, rs6589220, and rs3087967 as the best eQTL in the locus correlated with the expression level of *C11orf53*, *COLCA1*, and *COLCA2* genes in the colon transverse tissue (*P*_eQTL_ = 3.46 × 10^−16^, 2.71 × 10^−20^, and 3.98 × 10^−27^, respectively) (Figures 3(c)–3(e)). In addition, colocalization analysis identified a strong LD between the best eQTL (rs7130173) and the best eQTL (rs6589218, rs6589220, and rs3087967) (1.0 ≥ *r*^2^ ≥ 0.8) (Figures 3(c)–3(e)), and with PP4s for causality (*C11orf53*, PP4 = 0.99; *COLCA1*, PP4 = 0.99; and *COLCA2*, PP4 = 0.99, respectively), confirming that the significant CRC GWAS signal and colon transverse eQTL signals share the same variant at its locus.

Similarly, conditional and joint analyses showed that conditioning on *C11orf53* completely described the variance of the loci on chromosome 11 (beat SNP: rs7130173, *P*_GWAS_ = 2.52 × 10^−7^; conditioned on *C11orf53*, *P*_GWAS_ = 1). The fine-mapping findings revealed that the PIP values for *C11orf53*, *COLCA1*, and *COLCA2* were 0.36, 4.77 × 10^−3^, and 0.48, respectively. However, only *C11orf53* and *COLCA2* were included in the 90%-credible gene set.

### 3.8. Chromosome 15q13.3

The *GREM1-AS1* gene reached transcriptome-wide significance (*P*_TWAS_ = 1.54 × 10^−11^) and was the only significant association signal at this locus. Among this locus, rs1919360 was the most significant SNP associated with CRC, and the closest to the *GREM1* gene (OR = 1.51, *P*_GWAS_ = 1.91 × 10^−10^). The best eQTL in the locus associated with the expression level of *GREM1-AS1* was rs2611583 (*P*_eQTL_ = 4.42 × 10^−4^), which is shown to be weak LD with rs1919360 (0.2 ≥ *r*^2^ ≥ 0) (Figure 3(f)), while the fifth hypothesis was supported by formal Bayesian colocalization with a PP4 of 0.70 (Table 1), providing modest evidence that significant CRC GWAS signaling and colon sigmoid eQTL signaling share the same variants at their loci.

For the genomic locus 15q13.3, *GREM1-AS1* was included in the 90%-credible gene set with a PIP of 1 in the colon sigmoid (Table 1). At this locus, conditional and joint analyses found that *GREM1-AS1* explains 0.83 of the variance at its loci (best SNP: rs1919360, *P*_GWAS_ = 1.90 × 10^−10^; conditioned on *GREM1-AS1*, *P*_GWAS_ = 8.10 × 10^−3^) (Figure 3(f) and Figure S5d).

### 3.9. Summary Data-Based Mendelian Randomization Results

We applied SMR and HEIDI to investigate whether gene expression levels are mediated by genetic variation, testing causal associations between CRC susceptibility gene expression levels and CRC (Figure S6). We identified significantly associated gene expression levels in the colon transverse data from GTEx (v7) that passed the HEIDI test at chromosome 11q23.1 (Figure S7a) (*P*_SMR_ = 3.59 × 10^−6^), and there has been no substantial heterogeneity underlying the eQTL signals (*P*_HEIDI_ > 0.05). Also, this Mendelian randomization study showed a causal relationship between low *COLCA2* expression and CRC risk (Figure S7b).

We continued with SMR analyses of the eQTL summary data conducted by Westra et al. and Lloyd-Jones et al., respectively [34, 35]. However, after correcting for multiple testing, we were unable to detect any significant pleiotropic connection (Table S4).

### 3.10. Comparison with Previous Literature

Our TWAS study identified 6 genes significantly associated with CRC (*GREM1-AS1*, *C11orf53*, *POU5F1B*, *DUSP10*, *COLCA1*, and *COLCA2*) with a Bonferroni-corrected threshold of *P* < 1.11 × 10^−6^ (0.05/44,953). Compared with the previous largest TWAS study for CRC, which detected 25 associated genes with CRC risk at a Bonferroni-corrected threshold of *P* < 9.10 × 10^−6^ by MetaXcan software, we found that three of the significant TWAS genes (*C11orf53*, *COLCA1*, and *COLCA2*) were overlapped with the TWAS result performed by Guo et al. [36]. In addition, *SFMBT1* was almost close to the Bonferroni-corrected threshold in our research (*P* = 5.19 × 10^−6^), overlapping with the largest TWAS results for CRC. Obviously, in addition to the three significant genes mentioned above, we also identified three novel associations (*GREM1-AS1*, *POU5F1B*, and *DUSP10*). The above differences may be due to different GWAS summary statistics data or the type and quantity of SNP weight sets used, algorithm of the TWAS software and statistical thresholds.

### 3.11. Phenome-Wide Association Study

A PheWAS was performed for each best eQTL in 6 transcriptome-wide important features to further identify phenotypes that may be related or comorbid with CRC. In the process, we found that most of the eQTL were significantly associated with CRC, so we excluded the associated CRC traits to be able to effectively identify other phenotypes associated with them. Several best eQTL-related phenotypes were discovered to be strongly linked or comorbid with the risk of CRC, including bowel movement, alcohol consumption, C-C motif chemokine 22, family history of primary malignant neoplasm, cholelithiasis, and helicobacter pylori infection (Table S5) [14, 37–40].

## 4. Discussion

CRC is one of the most often diagnosed cancers, and it has a significant impact on cancer morbidity and mortality globally [41]. It is generally known that genetic factors play a significant role in the etiology of both familial and sporadic CRC [8, 42–44].

In this investigation, we first performed post-GWAS analyses of CRC GWAS summary data, including SNP annotation and gene-based and gene-set tests analysis using MAGMA, which was implemented in FUMA. In the follow-up gene-based analysis of GWAS, we identified 5 genes with significant association with CRC (*SMAD7*, *COLCA1*, *COLCA2*, *POU5F1B*, and *LAMA5*). In addition, gene-set analyses also identified neuroendocrine cell differentiation, LA metabolism, and other pathways associated with CRC, but they were not significant after multiple corrections. Second, we conducted a new TWAS on CRC of the European populations, which combined the CRC GWAS summary statistics and SNP weight sets to map four susceptibility loci on chromosomes 1q41, 8q24.21, 11q23.1, and 15q13.3. We confirmed three previously reported genes, including *C11orf53*, *COLCA1*, and *COLCA2*, and identified three novel association genes, *GREM1-AS1*, *POU5F1B*, and *DUSP10*.

We analyze the significant correlations further using a conditional analysis to evaluate if gene associations within the same genomic area are independent or if several genes are connected owing to correlated predicted expression. The six significant genes showed four independent associations with CRC, implying that probably half of the observed signal is influenced by LD and correlated predicted expression of nearby genes. We discovered that GWAS connections were explained to a large extent by TWAS associations when we compared the GWAS summary statistics before and after conditioning on significant TWAS correlations, implying the possibility of transcriptomic mediation of genetic risk for CRC.

The next colocalization analysis determined whether the genome-wide significant signal at the locus was driven by gene expression by testing whether the major variants of the GWAS and eQTL signals were identical. Specifically, Bayesian colocalization analyses were performed by COLOC package at the transcriptome-wide significant loci identified in this TWAS, and the PP4 of each gene sharing signal was calculated, providing evidence for whether the GWAS and eQTL signals share the same associations. We observed that no transcription-CRC signal was obtained from the same causal polymorphisms associated with SNP-CRC correlations, suggesting that most of the observed genes constituted linkage effects rather than pleiotropy. While these data show that transcription mediates the link between genetic vulnerability and CRC, neither TWAS nor colocalization can determine the causal. Therefore, we used SMR software to investigate the causal relationship between gene expression and CRC and only found that the *COLCA2* gene identified in colon transverse map to 11q23.1 with *COLCA1* and *C11orf53* closing the threshold of pSMR value, suggesting multiple causal signals at this locus.

We also utilized a TWAS fine-mapping approach called FOCUS to obtain additional insight into which genes are likely causative for CRC, fine-mapping causal genes from several TWAS correlations at a locus and highlighting a single feature as the causal relation. Fine-mapping of the corresponding genomic loci prioritized *GREM1-AS1* and *POU5F1B* in the 90%-credible gene set with a PIP of 1 in the colon sigmoid and colon transverse. For the genomic locus 11q23.1, *COLCA2* was included in the 90%-credible gene set with the highest PIP in the colon transverse tissue. Confusingly, the PIP value for the *DUSP10* gene was not available. Accordingly, we speculate that it may be due to the GWAS signal around the *DUSP10* gene not reaching the threshold of significance and not meeting the computational inclusion requirements of the FOCUS software [22]. The above findings largely reflected local patterns of LD and indicated the requirement for further functional identification at several of these complicated loci.

Based on the above-mentioned various analyses, we found that the *COLCA2* gene has an outstanding performance. *COLCA2* was recognized as a colorectal cancer-associated gene, like *COLCA1*, and they were coregulated genes transcribed from opposite strands of a region of chromosome 11q23 associated with colon cancer [45]. *COLCA2* is predominantly expressed in cells of epithelial, mesenchymal, and hematopoietic origin and has orthologs in a variety of mammals. Since *COLCA2* expression is reduced in tumor cells from subjects with higher risk alleles, *COLCA2* may play an important role in suppressing tumor formation in epithelial cells [45].

In compiling the results of this study's analysis, we also raise some limitations that were worth discussing. First, the limited sample size of the GTEX (v7) gene expression reference sample may have hampered the identification of subtle transcriptome effects on CRC heredity, emphasizing the need for bigger samples [21, 23]. Second, our TWAS methodology only examined cis-eQTL of gene expression and did not account for trans-eQTL effects [15, 34]. Future studies should devote resources to building larger gene expression reference plates to be able to investigate trans-QTL effects. Third, the samples in this study were exclusively European; therefore, the generalization of the findings to other ethnic groups was limited because of ethnic specificity.

## 5. Conclusion

In conclusion, we present the evidence for broad genomic and transcriptome alterations in colorectal cancer. Our study allows for the discovery of new connections as well as the elucidation of the genomic and transcriptome alterations that previously identified risk genes go through. We highlight genes that may be important for *SMAD7*, *LAMA5*, *GREM1-AS1*, *C11orf53*, *POU5F1B*, *DUSP10*, *COLCA1*, and *COLCA2*. These results suggest that GWAS and TWAS are effective statistical methods to observe small- and large-effect genes correlated with CRC, providing a testable target for further functional validation of CRC, assisting in the knowledge of the molecular basis of the disease.

## Figures and Tables

**Table 1:** List of the significant candidate genes identified by TWAS for colorectal cancer.

Genes	Tissues	Cytogenetic band	Best eQTLs	Best SNPs	*Z*-scores	*P*_TWAS_	PP4	*P*_Permutation_	PIPs	90% credible set^∗^
*GREM1-AS1*	Colon sigmoid	15q13.3	rs2611583	rs1919360	6.74	1.54 × 10^−11^	0.70	0.002	1	1
*C11orf53*	Colon transverse	11q23.1	rs6589218	rs7130173	-5.14	2.80 × 10^−7^	0.99	0.002	0.36	1
*POU5F1B*	Colon transverse	8q24.21	rs7014346	rs6983267	-5.05	4.32 × 10^−7^	0.73	0.003	1	1
*DUSP10*	Colon sigmoid	1q41	rs6695584	rs12125368	4.92	8.72 × 10^−7^	0.52	0.003	NA	NA
*COLCA1*	Colon transverse	11q23.1	rs6589220	rs7130173	-4.88	1.08 × 10^−6^	0.99	0.019	0.00477	0
*COLCA2*	Colon transverse	11q23.1	rs3087967	rs7130173	-4.87	1.11 × 10^−6^	0.99	0.001	0.48	1

FUSION, FOCUS, and colocalization were used to identify genes associated with CRC. And 1000 Genomes Phase 3 (Europe, *N* = 489) data were used as the LD reference panel. *P*_TWAS_ is the *P* value of TWAS result; TWAS: transcriptome-wide association study; PP4 shared eQTL and GWAS associations; *P*_permutation_ was the result of each of the significant features using FUSION. PIP: posterior inclusion probability of fine-mapping; ∗: 1 means included in the 90% credible set; 0 means not included in the 90% credible set of FOCUS. NA: not available.

## Data Availability

The original contributions presented in the study are included in the article/supplementary material; further inquiries can be directed to the corresponding author.

## Abbreviations

CRC: Colorectal cancer
eQTL: Expression quantitative loci
FOCUS: Fine-mapping Of CaUsal gene Sets
GWAS: Genome-wide association studies
GTEx: Genotype-Tissue Expression
HEIDI: Heterogeneity independent instrument
LD: Linkage disequilibrium
Mb: Megabase
MR: Mendelian randomization
OR: Odds ratio
pheWAS: Phenome-wide association study
PIP: Posterior inclusion probability
PP: Posterior probability
SNPs: Single nucleotide polymorphisms
SMR: Summary data-based Mendelian randomization
TWAS: Transcriptome-wide association studies.

## Supplementary Materials

Supplementary Materials Table S1: functional consequences of SNPs on genes. Table S2: summary statistics for significant genes (*P* < 2.60*E* − 6) identified in the gene-based. Table S3: results of the gene-set analyses. Table S4: the top ten probes were identified in the SMR analysis for 5 eQTL summary data. Table S5: phenotypes associated with top eQTLs derived from TWAS. Figure S1: Manhattan plot and quantile-quantile plot of the genome-wide *P* values in the GWAS analysis. Figure S2: functional consequences of SNPs on genes. Figure S3: regional plots for associations in the regions surrounding the rs6983267, rs58658771, and rs6507874 in the GWAS analysis. Figure S4: gene-based genome-wide analysis for CRC by MAGMA to each locus in the GWAS analysis. Figure S5: conditional transcriptome-wide association study analysis for loci showing statistically significant associations with multiple genes. Figure S6: schematic diagram of the summary data-based Mendelian randomization model. Figure S7: prioritizing genes at the COLCA2 locus for CRC.
