# Supplementary material for: Genome-Wide Association and Transcriptome-Wide Association Studies Identify Novel Susceptibility Genes Contributing to Colorectal Cancer
Source: J Immunol Res. 2022 Jul 1;2022:5794055. doi: 10.1155/2022/5794055 (PMC9270168; doi:10.1155/2022/5794055)
Supplement: Supplementary Materials — Table S1: functional consequences of SNPs on genes. Table S2: summary statistics for significant genes (P < 2.60E − 6) identified in the gene-based. Table S3: results of the gene-set analyses. Table S4: the top ten probes were identified in the SMR analysis for 5 eQTL summary data. Table S5: phenotypes associated with top eQTLs derived from TWAS. Figure S1: Manhattan plot and quantile-quantile plot of the genome-wide P values in the GWAS analysis. Figure S2: functional consequences of SNPs on genes. Figure S3: regional plots for associations in the regions surrounding the rs6983267, rs58658771, and rs6507874 in the GWAS analysis. Figure S4: gene-based genome-wide analysis for CRC by MAGMA to each locus in the GWAS analysis. Figure S5: conditional transcriptome-wide association study analysis for loci showing statistically significant associations with multiple genes. Figure S6: schematic diagram of the summary data-based Mendelian randomization model. Figure S7: prioritizing genes at the COLCA2 locus for CRC. [file 5794055.f1.docx]

Supplementary Material

# Supplementary Tables

**Table S1** Functional consequences of SNPs on genes.

| **Catalog** | **Ref.count** | **Ref.prop** | **Counts** | **Prop.** | **Enrichment** | ***P*_Fisher_** |
| --- | --- | --- | --- | --- | --- | --- |
| UTR3 | 233,824 | 9.32E-03 | 20 | 1.54E-01 | 1.65E+01 | 1.54E-18 |
| UTR5 | 71,546 | 2.85E-03 | 0 | 0 | 0 | 1 |
| Downstream | 284,177 | 1.13E-02 | 1 | 7.69E-03 | 6.79E-01 | 1 |
| Exonic | 254,736 | 1.02E-02 | 3 | 2.31E-02 | 2.27E+00 | 1.47E-01 |
| Intergenic | 11,684,523 | 4.66E-01 | 20 | 1.54E-01 | 3.30E-01 | 7.68E-14 |
| Intronic | 9,137,749 | 3.64E-01 | 42 | 3.23E-01 | 8.87E-01 | 3.63E-01 |
| ncRNA_exonic | 259,951 | 1.04E-02 | 3 | 2.31E-02 | 2.23E+00 | 1.53E-01 |
| ncRNA_intronic | 2,884,355 | 1.15E-01 | 41 | 3.15E-01 | 2.74E+00 | 9.39E-10 |
| ncRNA_splicing | 1,313 | 5.23E-05 | 0 | 0 | 0 | 1 |
| Splicing | 2,830 | 1.13E-04 | 0 | 0 | 0 | 1 |
| Upstream | 266,686 | 1.06E-02 | 0 | 0 | 0 | 6.52E-01 |
| Enrichment of functional consequences of SNPs was tested against the reference panel population of the 1000 Genomes Phase 3 European. All SNPs that are in LD with one of the independent significant SNPs are annotated by FUMA (https://fuma.ctglab.nl/). Enrichment value was computed as (proportion of SNPs with an annotation) /(proportion of SNPs with an annotation relative to all available SNPs in the reference panel). Fisher's exact test (two sides) was performed for each annotation as above. SNP, single-nucleotide polymorphism; Ref, reference; Prop, proportion; ncRNA, non-coding RNA. | | | | | | |
|  |  |  |  |  |  |  |
|  |  |  |  |  |  |  |
|  |  |  |  |  |  |  |
|  |  |  |  |  |  |  |
|  |  |  |  |  |  |  |
|  |  |  |  |  |  |  |

**Table S2.** Summary statistics for significant genes (*P* < 2.60E-6) identified in the gene-based analysis.

| **Genes** | **Chr.** | **Start** | **Stop** | **No. of SNPs** | **NPARAM** | **N** | **Z_STAT_** | ***P*** | **Symbols** |
| --- | --- | --- | --- | --- | --- | --- | --- | --- | --- |
| ENSG00000101665 | 18 | 46445223 | 46479081 | 192 | 54 | 387,318 | 5.7222 | 5.26E-09 | *SMAD7* |
| ENSG00000196167 | 11 | 111163114 | 111177770 | 84 | 26 | 387,318 | 5.1489 | 1.31E-07 | *COLCA1* |
| ENSG00000214290 | 11 | 111167565 | 111180460 | 73 | 25 | 387,318 | 5.0787 | 1.90E-07 | *COLCA2* |
| ENSG00000212993 | 8 | 128424535 | 128433311 | 62 | 14 | 387,318 | 5.0766 | 1.92E-07 | *POU5F1B* |
| ENSG00000130702 | 20 | 60882011 | 60944368 | 576 | 71 | 387,318 | 4.5737 | 2.40E-06 | *LAMA5* |
| Gene-based analysis was performed by MAGMA (https://ctg.cncr.nl/software/magma), all SNPs located within genes were mapped to 19,252 protein-coding genes. Genomic positions are based on NCBI Build 37. No. of SNPs: the number of SNPs annotated to that gene. NPARAM: the number of relevant parameters used in the model. N: the sample size used when analyzing that gene. Z_STAT_: the Z-value for the gene, based on its (permutation) *P*-value. *P*: the gene *P*-value, using asymptotic sampling distribution. | | | | | | | | | |
|  |  |  |  |  |  |  |  |  |  |
|  |  |  |  |  |  |  |  |  |  |
|  |  |  |  |  |  |  |  |  |  |
|  |  |  |  |  |  |  |  |  |  |

**Table S3.** Results of the gene-set analyses.

| **No.** | **Gene Set** | **No. of genes** | **Beta** | **SE** | ***P*** | ***P*_bon_** |
| --- | --- | --- | --- | --- | --- | --- |
| 1 | GO BP: neuroendocrine cell differentiation | 8 | 1.56 | 0.35 | 5.28E-06 | 0.08 |
| 2 | GO BP: renal vesicle development | 19 | 0.90 | 0.22 | 1.33E-05 | 0.21 |
| 3 | GO BP: mesonephros development | 98 | 0.37 | 0.09 | 1.37E-05 | 0.21 |
| 4 | Curated gene sets: fukushima tnfsf11 targets | 14 | 1.04 | 0.25 | 1.88E-05 | 0.29 |
| 5 | Curated gene sets: linoleic acid (LA) metabolism | 7 | 1.32 | 0.32 | 2.28E-05 | 0.35 |
| 6 | GO BP: metanephric nephron morphogenesis | 25 | 0.73 | 0.19 | 5.12E-05 | 0.79 |
| 7 | GO BP: metanephric renal vesicle morphogenesis | 15 | 0.96 | 0.25 | 5.50E-05 | 0.85 |
| 8 | GO BP: renal vesicle formation | 8 | 1.25 | 0.32 | 5.58E-05 | 0.86 |
| 9 | GO BP: negative regulation of pathway restricted smad protein phosphorylation | 11 | 1.04 | 0.27 | 5.58E-05 | 0.86 |
| 10 | GO BP: negative regulation of mononuclear cell migration | 8 | 1.26 | 0.33 | 6.37E-05 | 0.99 |
| MAGMA gene-set analysis was performed for curated gene sets and GO terms obtained from MsigDB. The table displays either significant gene sets with *P*_bon_ < 0.05 or top 10 gene sets when there are less than 10 significant gene sets. Note that MAGMA gene-set analyses used the full distribution of SNP p-values and were different from a pathway enrichment test as implemented in GENE2FUNC that only tests for enrichment of prioritized genes. No., number. BP, biological process. *P*_bon,_ the gene-set *P*-value, after Bonferroni correction for multiple testing. | | | | | | |
|  |  |  |  |  |  |  |
|  |  |  |  |  |  |  |
|  |  |  |  |  |  |  |

**Table S4.** The top ten probes were identified in the SMR analysis for 5 eQTL summary data.

| eQTL data | probeID | Chr. | Gene | topSNP | p_GWAS | p_eQTL | b_SMR | se_SMR | p_SMR | p_HEIDI | nsnp_HEIDI |
| --- | --- | --- | --- | --- | --- | --- | --- | --- | --- | --- | --- |
| cage_eqtl_data | ILMN_2092756 | 11 | *TMEM109* | rs12576127 | 6.27E-05 | 2.85E-28 | 0.278768 | 0.0739856 | 1.65E-04 | 3.69E-01 | 20 |
|  | ILMN_1789106 | 1 | *IPP* | rs28361459 | 3.81E-04 | 2.09E-50 | -0.18273 | 0.0528568 | 5.46E-04 | 3.74E-01 | 15 |
|  | ILMN_1786176 | 19 | *CD37* | rs10412811 | 4.06E-04 | 6.84E-59 | 0.168512 | 0.0488551 | 5.62E-04 | 1.15E-02 | 20 |
|  | ILMN_1654370 | 1 | *TESK2* | rs10890329 | 3.40E-04 | 5.13E-26 | 0.265593 | 0.078263 | 6.90E-04 | 2.93E-01 | 20 |
|  | ILMN_1682781 | 19 | *TEAD2* | rs7256984 | 6.19E-04 | 8.86E-60 | 0.161244 | 0.0480127 | 7.84E-04 | 5.59E-01 | 20 |
|  | ILMN_1675156 | 1 | *CDC42* | rs2473290 | 8.59E-04 | 7.00E-118 | -0.109315 | 0.0331474 | 9.74E-04 | 1.78E-02 | 20 |
|  | ILMN_1714438 | 1 | *MUTYH* | rs12139364 | 8.78E-04 | 7.71E-85 | -0.133616 | 0.0407948 | 1.06E-03 | 2.97E-01 | 18 |
|  | ILMN_1723287 | 19 | *SLC6A16* | rs10422677 | 3.33E-04 | 1.39E-14 | -0.365605 | 0.112389 | 1.14E-03 | 4.65E-02 | 20 |
|  | ILMN_2382657 | 12 | *ARHGAP9* | rs899653 | 6.41E-04 | 7.19E-25 | 0.312883 | 0.0962708 | 1.15E-03 | 1.17E-02 | 20 |
|  | ILMN_1757272 | 8 | *THAP1* | rs112061290 | 5.67E-04 | 1.81E-19 | -0.41081 | 0.127768 | 1.30E-03 | 2.88E-01 | 20 |
| westra_eqtl_hg19 | ILMN_2092756 | 11 | *TMEM109* | rs2074418 | 6.98E-05 | 4.35E-27 | 0.408211 | 0.109297 | 1.88E-04 | 3.98E-01 | 20 |
|  | ILMN_1774604 | 2 | *PNKD* | rs4672884 | 2.85E-04 | 2.80E-201 | 0.143416 | 0.0398583 | 3.20E-04 | 9.55E-01 | 20 |
|  | ILMN_1731275 | 16 | *DPEP3* | rs255052 | 2.77E-04 | 5.64E-75 | -0.229789 | 0.0644247 | 3.61E-04 | 1.52E-03 | 11 |
|  | ILMN_1691410 | 10 | *BAMBI* | rs1775908 | 2.56E-04 | 2.46E-57 | 0.259541 | 0.0728882 | 3.70E-04 | 5.82E-01 | 20 |
|  | ILMN_1906437 | 12 | *HS.569104* | rs11170800 | 1.74E-04 | 5.51E-19 | -0.466937 | 0.135045 | 5.45E-04 | 7.73E-01 | 12 |
|  | ILMN_1681644 | 11 | *BIRC3* | rs2155587 | 4.75E-04 | 6.03E-64 | 0.15787 | 0.0460833 | 6.13E-04 | 5.28E-01 | 5 |
|  | ILMN_1714438 | 1 | *MUTYH* | rs12139364 | 8.78E-04 | 1.03E-154 | -0.146995 | 0.0445898 | 9.79E-04 | 2.22E-01 | 20 |
|  | ILMN_1723287 | 19 | *SLC6A16* | rs10414921 | 3.22E-04 | 1.08E-15 | -0.493924 | 0.150502 | 1.03E-03 | 6.13E-02 | 16 |
|  | ILMN_1652198 | 7 | *CCM2* | rs4724354 | 1.08E-03 | 5.03E-308 | -0.105845 | 0.0325164 | 1.13E-03 | 4.10E-01 | 20 |
|  | ILMN_1780058 | 1 | *DEGS1* | rs10916489 | 1.07E-03 | 5.27E-179 | 0.135558 | 0.0418683 | 1.20E-03 | 9.12E-03 | 20 |
| Colon_Sigmoid | ENSG00000218510.3 | 1 | *LINC00339* | rs11586488 | 7.36E-04 | 2.13E-71 | 0.0605696 | 0.018285 | 9.25E-04 | 3.31E-01 | 20 |
|  | ENSG00000226332.2 | 20 | *RP11-157P1.4* | rs2427283 | 1.50E-04 | 1.01E-11 | -0.264332 | 0.0799493 | 9.46E-04 | 4.40E-01 | 20 |
|  | ENSG00000268686.1 | 19 | *AC010524.2* | rs7254187 | 4.61E-04 | 1.83E-19 | 0.0934232 | 0.0286014 | 1.09E-03 | 6.06E-01 | 20 |
|  | ENSG00000130270.12 | 19 | *ATP8B3* | rs7250872 | 2.59E-04 | 7.92E-13 | 0.15294 | 0.0469892 | 1.13E-03 | 7.36E-02 | 20 |
|  | ENSG00000198035.9 | 10 | *AGAP9* | rs72472022 | 3.65E-04 | 7.24E-14 | -0.138484 | 0.043013 | 1.28E-03 | 6.99E-02 | 8 |
|  | ENSG00000161692.13 | 17 | *DBF4B* | rs34839354 | 9.99E-04 | 9.11E-35 | 0.106735 | 0.0335919 | 1.49E-03 | 1.98E-01 | 20 |
|  | ENSG00000104901.2 | 19 | *DKKL1* | rs7248176 | 6.52E-04 | 2.62E-15 | 0.102589 | 0.0326994 | 1.70E-03 | 4.20E-01 | 20 |
|  | ENSG00000132031.8 | 2 | *MATN3* | rs11694716 | 9.87E-04 | 1.81E-15 | 0.12581 | 0.0412706 | 2.30E-03 | 2.06E-01 | 20 |
|  | ENSG00000261338.1 | 2 | *RP11-378A13.1* | rs12999734 | 1.63E-03 | 1.65E-31 | 0.0841309 | 0.027678 | 2.37E-03 | 6.14E-01 | 20 |
|  | ENSG00000179296.9 | 10 | *CTGLF12P* | rs7084135 | 3.25E-04 | 2.12E-08 | 0.177617 | 0.0585998 | 2.44E-03 | 9.25E-02 | 5 |
| Colon_Transverse | ENSG00000214290.3 | 11 | *COLCA2* | rs3087967 | 3.68E-07 | 3.14E-28 | -0.173111 | 0.0373596 | 3.59E-06 | 9.25E-01 | 20 |
|  | ENSG00000196167.5 | 11 | *COLCA1* | rs6589220 | 2.95E-06 | 2.71E-20 | -0.204449 | 0.0490194 | 3.04E-05 | 7.63E-01 | 20 |
|  | ENSG00000150750.6 | 11 | *C11orf53* | rs11213823 | 3.07E-06 | 1.05E-17 | -0.269113 | 0.0655712 | 4.06E-05 | 6.07E-01 | 20 |
|  | ENSG00000163935.9 | 3 | *SFMBT1* | rs2581817 | 2.90E-06 | 7.10E-14 | 0.2559 | 0.0647406 | 7.73E-05 | 3.07E-01 | 20 |
|  | ENSG00000110107.4 | 11 | *PRPF19* | rs10897126 | 5.75E-05 | 4.92E-12 | -0.35083 | 0.100833 | 5.03E-04 | 6.62E-01 | 20 |
|  | ENSG00000218510.3 | 1 | *LINC00339* | rs11801382 | 4.07E-04 | 2.05E-53 | 0.0799685 | 0.0231898 | 5.64E-04 | 1.39E-01 | 20 |
|  | ENSG00000170632.9 | 7 | *ARMC10* | rs6465890 | 8.42E-05 | 8.16E-13 | 0.292571 | 0.0848533 | 5.65E-04 | 1.49E-01 | 4 |
|  | ENSG00000115718.13 | 2 | *PROC* | rs2069933 | 4.98E-04 | 2.11E-18 | 0.159607 | 0.0493254 | 1.21E-03 | 1.19E-01 | 20 |
|  | ENSG00000123384.9 | 12 | *LRP1* | rs7398375 | 2.07E-04 | 2.24E-10 | 0.366986 | 0.114666 | 1.37E-03 | 8.11E-01 | 11 |
|  | ENSG00000161692.13 | 17 | *DBF4B* | rs16970944 | 1.13E-03 | 2.02E-36 | 0.125942 | 0.0399138 | 1.60E-03 | 3.07E-02 | 20 |
| Whole_Blood | ENSG00000246228.2 | 8 | *CASC8* | rs74673821 | 5.10E-05 | 7.28E-38 | 0.106973 | 0.0277595 | 1.16E-04 | 7.37E-04 | 20 |
|  | ENSG00000095739.7 | 10 | *BAMBI* | rs1775906 | 2.18E-04 | 1.12E-22 | 0.142783 | 0.0412407 | 5.36E-04 | 6.08E-01 | 19 |
|  | ENSG00000127838.9 | 2 | *PNKD* | rs10203039 | 1.82E-04 | 4.35E-19 | 0.27078 | 0.0782989 | 5.44E-04 | 4.55E-01 | 20 |
|  | ENSG00000054277.8 | 1 | *OPN3* | rs658729 | 1.64E-04 | 2.26E-13 | 0.261164 | 0.077935 | 8.05E-04 | 3.75E-01 | 17 |
|  | ENSG00000050767.11 | 5 | *COL23A1* | rs684612 | 1.81E-04 | 2.96E-13 | -0.219622 | 0.0657856 | 8.42E-04 | 1.67E-01 | 19 |
|  | ENSG00000218510.3 | 1 | *LINC00339* | rs12097775 | 3.54E-04 | 4.39E-19 | 0.203448 | 0.0613472 | 9.12E-04 | 7.78E-03 | 20 |
|  | ENSG00000259235.1 | 15 | *RP11-605F22.2* | rs74012001 | 1.03E-03 | 6.98E-94 | -0.0749601 | 0.0231168 | 1.18E-03 | 8.84E-01 | 20 |
|  | ENSG00000074803.13 | 15 | *SLC12A1* | rs74012001 | 1.03E-03 | 2.09E-77 | -0.0706714 | 0.0218533 | 1.22E-03 | 7.85E-01 | 20 |
|  | ENSG00000170632.9 | 7 | *ARMC10* | rs6465890 | 8.42E-05 | 2.33E-08 | 0.601426 | 0.186982 | 1.30E-03 | NA | NA |
|  | ENSG00000128951.9 | 15 | *DUT* | rs76749119 | 1.07E-03 | 1.37E-59 | -0.109793 | 0.0342098 | 1.33E-03 | 7.29E-01 | 20 |
| SMR and HEIDI were performed to test the causal relationship between colorectal cancer susceptibility gene expression levels and CRC using pooled statistics from GWAS CRC and eQTL weighting data (colon sigmoid, colon transverse colon, whole blood from GTEx [v7] and Westra, CAGE eQTL summary data). Chr, chromosome. eQTL, expression quantitative trait locus. HEIDI, heterogeneity independent instruments. SMR, summary data-based Mendelian randomization. | | | | | | | | | | | |
|  |  |  |  |  |  |  |  |  |  |  |  |

**Table S5.** Phenotypes associated with top eQTLs derived from TWAS.

| Best eQTL | Phenotypes (excluding CRC) | *P*-value |
| --- | --- | --- |
| rs2611583 | Illnesses of mother: Stroke | 4.43E-04 |
|  | CTSZ - Cathepsin Z | 5.39E-04 |
|  | EPHA3 - Ephrin type-A receptor 3 | 6.41E-04 |
|  | Total IgM levels | 1.31E-03 |
|  | CGA TSHB - Thyroid Stimulating Hormone | 1.63E-03 |
|  | Alcohol dependence | 1.85E-03 |
|  | Sensitivity / hurt feelings (HURT) | 3.11E-03 |
|  | Menstruation quality of life impact: Bowel movement | 3.41E-03 |
|  | Sensitivity / hurt feelings | 3.44E-03 |
|  | Left precuneus | 3.93E-03 |
| rs6589218 | Diagnoses - main ICD10: D12 Benign neoplasm of colon, rectum, anus and anal canal | 1.80E-13 |
|  | Alcohol consumption (dichotomous, male) | 2.49E-04 |
|  | CCL22 - C-C motif chemokine 22 | 3.42E-04 |
|  | Diagnoses - secondary ICD10: Z80 Family history of primary malignant neoplasm | 4.39E-04 |
|  | Standing height | 5.27E-04 |
|  | Posterior thalamic radiation (include optic radiation) mode of anisotropy | 6.59E-04 |
|  | Diagnoses - main ICD10: K80 Cholelithiasis | 7.77E-04 |
|  | Helicobacter pylori IgG levels | 9.07E-04 |
|  | CD33 - Myeloid cell surface antigen CD33 | 9.92E-04 |
|  | TNFRSF8 - Tumor necrosis factor receptor superfamily member 8 | 1.49E-03 |
| rs7014346 | Cancer register - Histology of cancer tumour: Adenocarcinoma, NOS | 1.83E-15 |
|  | Diagnoses - main ICD10: D12 Benign neoplasm of colon, rectum, anus and anal canal | 1.48E-08 |
|  | Illnesses of father: Prostate cancer | 1.26E-06 |
|  | Number of self-reported cancers | 2.73E-05 |
|  | Generalized epilepsy with tonic-clonic seizures | 5.00E-05 |
|  | Illnesses of father: Bowel cancer | 7.47E-05 |
|  | Illnesses of mother: Bowel cancer | 2.26E-04 |
|  | Lipid::Fatty acid, dicarboxylate::3-carboxy-4-methyl-5-propyl-2-furanpropanoate (CMPF) | 3.16E-04 |
|  | Wheeze or whistling in the chest in last year | 4.47E-04 |
|  | Cancer (diagnosed by doctor) | 4.66E-04 |
| rs6695584 | Impedance measures - Arm fat percentage (left) | 8.50E-06 |
|  | Impedance measures - Arm fat percentage (right) | 1.71E-05 |
|  | Illnesses of father: Bowel cancer | 1.86E-04 |
|  | Impedance measures - Trunk fat percentage | 2.79E-04 |
|  | Diagnoses - main ICD10: D12 Benign neoplasm of colon, rectum, anus and anal canal | 3.07E-04 |
|  | Body Mass Index | 3.71E-04 |
|  | Body Mass Index (female) | 7.35E-04 |
|  | Legs-leg fat ratio (female) | 7.82E-04 |
|  | Mean platelet volume (two-way meta) | 8.37E-04 |
|  | Impedance measures - Arm fat mass (left) | 1.03E-03 |
| rs6589220 | Diagnoses - main ICD10: D12 Benign neoplasm of colon, rectum, anus and anal canal | 8.71E-14 |
|  | Alcohol consumption (dichotomous, male) | 2.02E-04 |
|  | Standing height | 4.11E-04 |
|  | Cognitive performance | 8.18E-04 |
|  | Cancer register - Type of cancer: ICD10: C44 Other and unspecified malignant neoplasm of skin | 1.25E-03 |
|  | Cancer register - Histology of cancer tumour: Basal cell carcinoma, NOS | 1.75E-03 |
|  | Mouth/teeth dental problems: Loose teeth | 1.94E-03 |
|  | Height | 2.00E-03 |
|  | College completion | 2.60E-03 |
|  | Cancer register - Histology of cancer tumour: Adenocarcinoma, NOS | 2.97E-03 |
| rs3087967 | Diagnoses - main ICD10: D12 Benign neoplasm of colon, rectum, anus and anal canal | 2.86E-15 |
|  | Alcohol consumption (dichotomous, male) | 1.83E-04 |
|  | Standing height | 1.97E-04 |
|  | Cognitive performance | 2.08E-04 |
|  | CD33 - Myeloid cell surface antigen CD33 | 2.77E-04 |
|  | CCL22 - C-C motif chemokine 22 | 3.91E-04 |
|  | Cancer register - Histology of cancer tumour: Adenocarcinoma, NOS | 4.37E-04 |
|  | Cancer register - Type of cancer: ICD10: C44 Other and unspecified malignant neoplasm of skin | 4.38E-04 |
|  | PROS1 - Vitamin K-dependent protein S | 8.89E-04 |
|  | Had menopause (female) | 9.31E-04 |
| To understand phenotypes that may be associated or co-morbid with ADHD, a pheWAS was done for each eQTL. CRC, colorectal cancer. eQTL, expression quantitative trait loci. TWAS, transcriptome-wide association study. | | |
|  |  |  |

1. **Supplementary Figures**


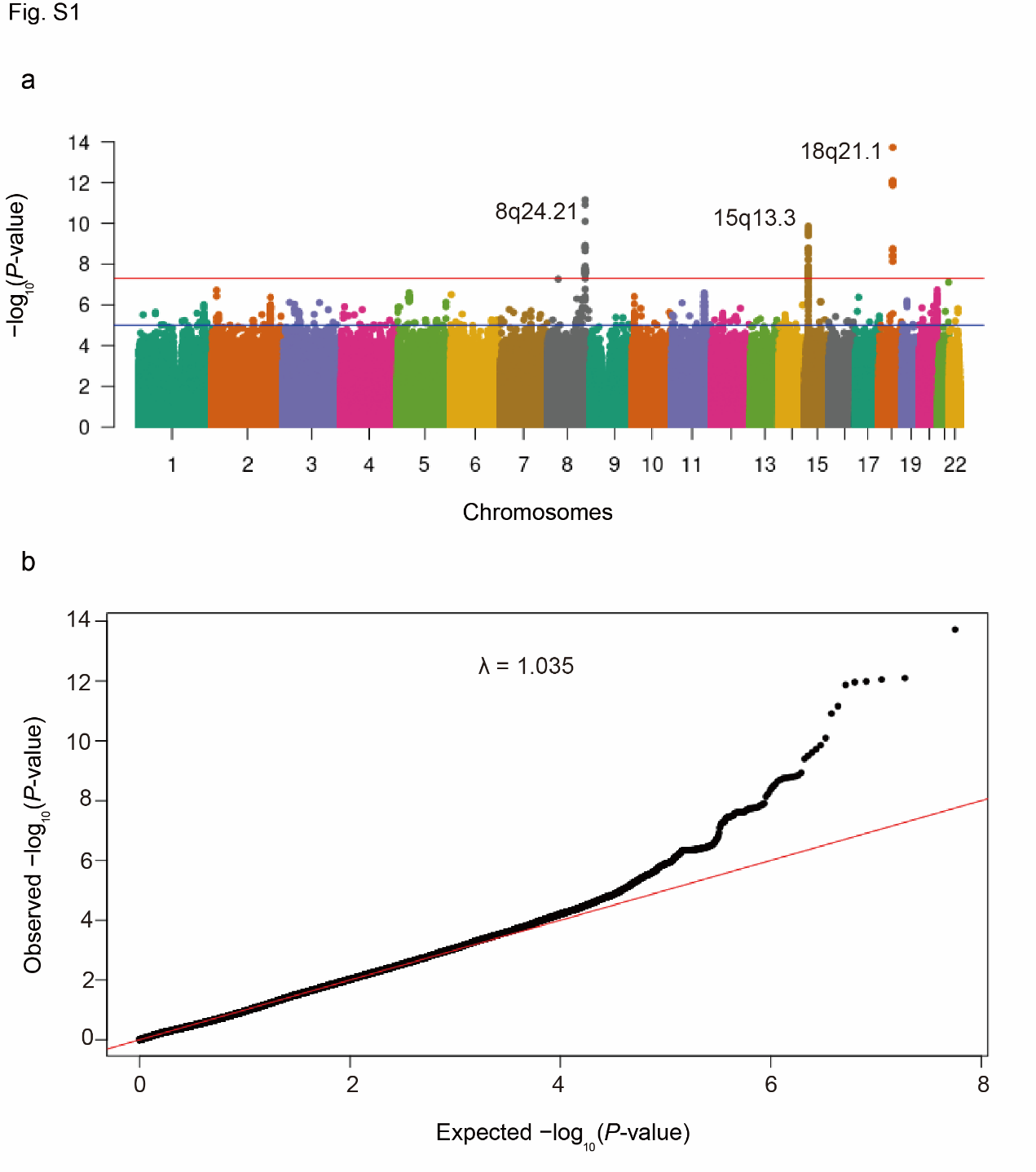


**Fig. S1 Manhattan plot and Quantile-quantile plot of the genomewide *P* values in the GWAS analysis.** (**a**) The Manhattan plot showed the genome-wide association statistics from the GWAS analysis. The x-axis represents the genomic position (based on NCBI Build 37), and the y-axis shows the -log_10_ (*P*). The red dashed line indicates the genome-wide significance threshold of *P* = 5 × 10^-8^. The blue dashed line indicates the suggestive significance threshold of *P* = 1 × 10^-5^. (**b**) The quantile-quantile plot. The red line represents the null hypothesis of no true association. The black dot with gradient λ (inflation coefficient) is fitted to the lower 90% of the distribution of the observed test statistics. The value of the inflation factor is 1.035.


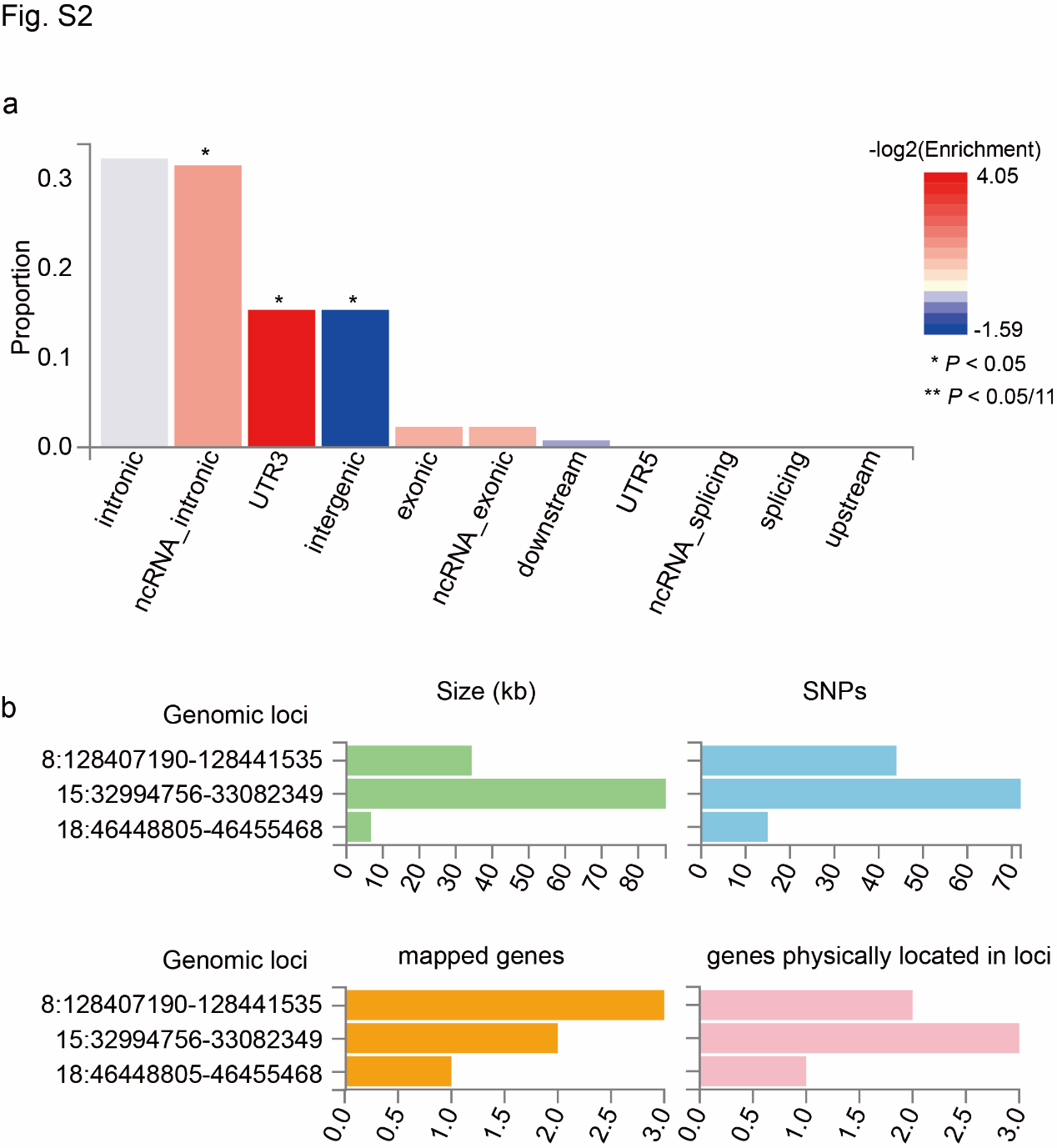


**Fig. S2 Functional consequences of SNPs on genes.** Enrichment of functional consequences of SNPs was tested against the reference panel population of the 1000 Genomes Phase 3 European. All SNPs that are in LD with one of the independent significant SNPs are annotated by FUMA (https://fuma.ctglab.nl/). Enrichment value was computed as (proportion of SNPs with an annotation) /(proportion of SNPs with an annotation relative to all available SNPs in the reference panel). Fisher's exact test (two sides) was performed for each annotation as above and then the enrichment levels were log2 transformed. (**b**) Summary per genomic risk locus. After functional annotation analyses, we annotated 131 candidate SNPs that passed the gene-wide significance threshold (*P* < 5.00 × 10^-8^), 3 independent lead SNPs were identified located at 3genomic risk loci. SNP, single-nucleotide polymorphism; kb, kilobase; ncRNA, non-coding RNA.

**
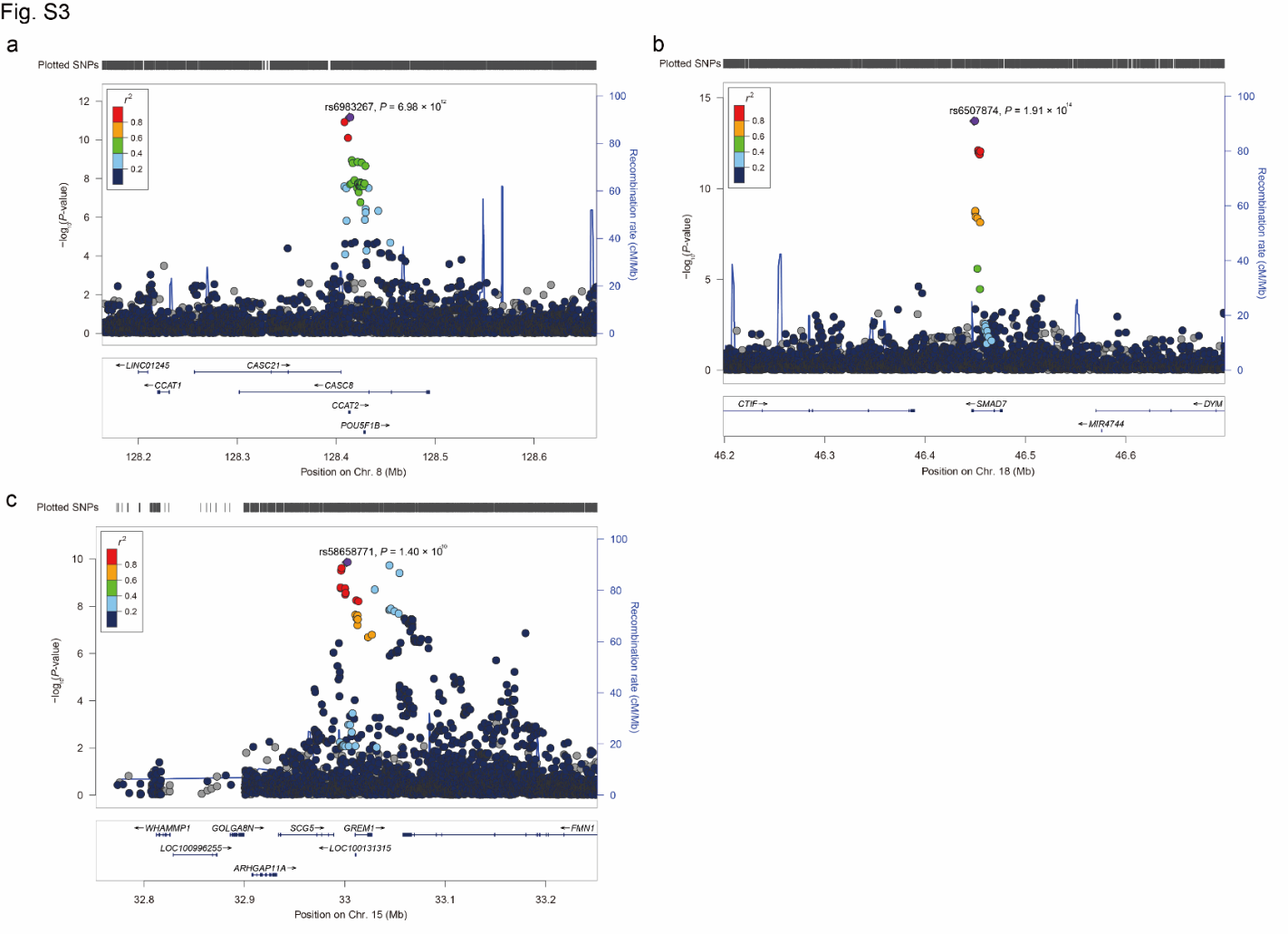
**

**Fig. S3 Regional plots for associations in the regions surrounding the rs6983267, rs58658771 and rs6507874 in the GWAS analysis**. Results are shown for SNPs in the region 250 kb up-or downstream of the marker SNP rs6983267 (**a**), rs58658771 (**b**) and rs6507874 (**c**). Genomic positions are based on NCBI Build 37. The *P* values of the SNPs in these four regions in the GWAS analysis are shown and the *P* values of rs6983267, rs58658771 and rs6507874 are shown as purple dots. The linkage disequilibrium (LD) values (*r*^2^) to the index SNPs (rs6983267, rs58658771 and rs6507874) for the other SNPs are indicated by marker color. Red signifies *r*^2^ ≥ 0.8, with orange 0.6 ≤ *r*^2^ < 0.8, green 0.4 ≤ *r*^2^ < 0.6, light blue 0.2 ≤ *r*^2^ < 0.4, and blue *r*^2^ < 0.2. The estimated recombination rates in the European population (from the 1000 Genomes Project, November, 2014) are plotted in light blue. GWAS, genome-wide association study; LD, linkage disequilibrium; SNP, single nucleotide polymorphism.


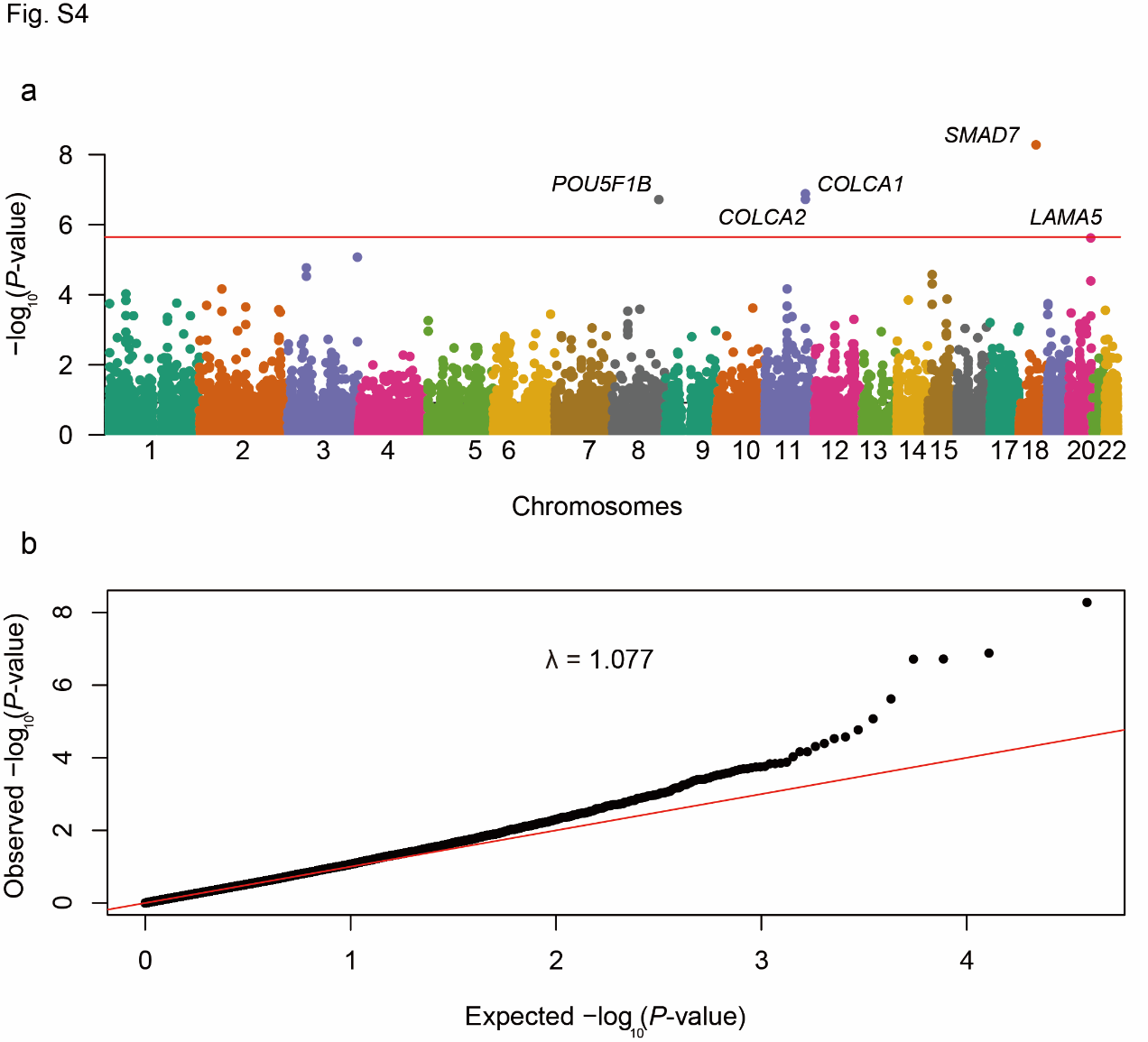


**Fig. S4 Gene-based genome-wide analysis for CRC by MAGMA to each locus in the GWAS analysis.** (**a**) Significant genes in the gene-based association test in MAGMA after Bonferroni correction (*P* < 0.05/19252 = 2.60 × 10^-6^). The x-axis represents the chromosome number, and the y-axis shows the negative log10-transformed gene-based *P*-value. The top 5 most significant genes are annotated with the corresponding gene symbols. (**b**) The quantile-quantile plot. The red line represents the null hypothesis of no true association. The black dot with gradient λ (inflation coefficient) is fitted to the lower 90% of the distribution of the observed test statistics. The value of the inflation factor is 1.077.


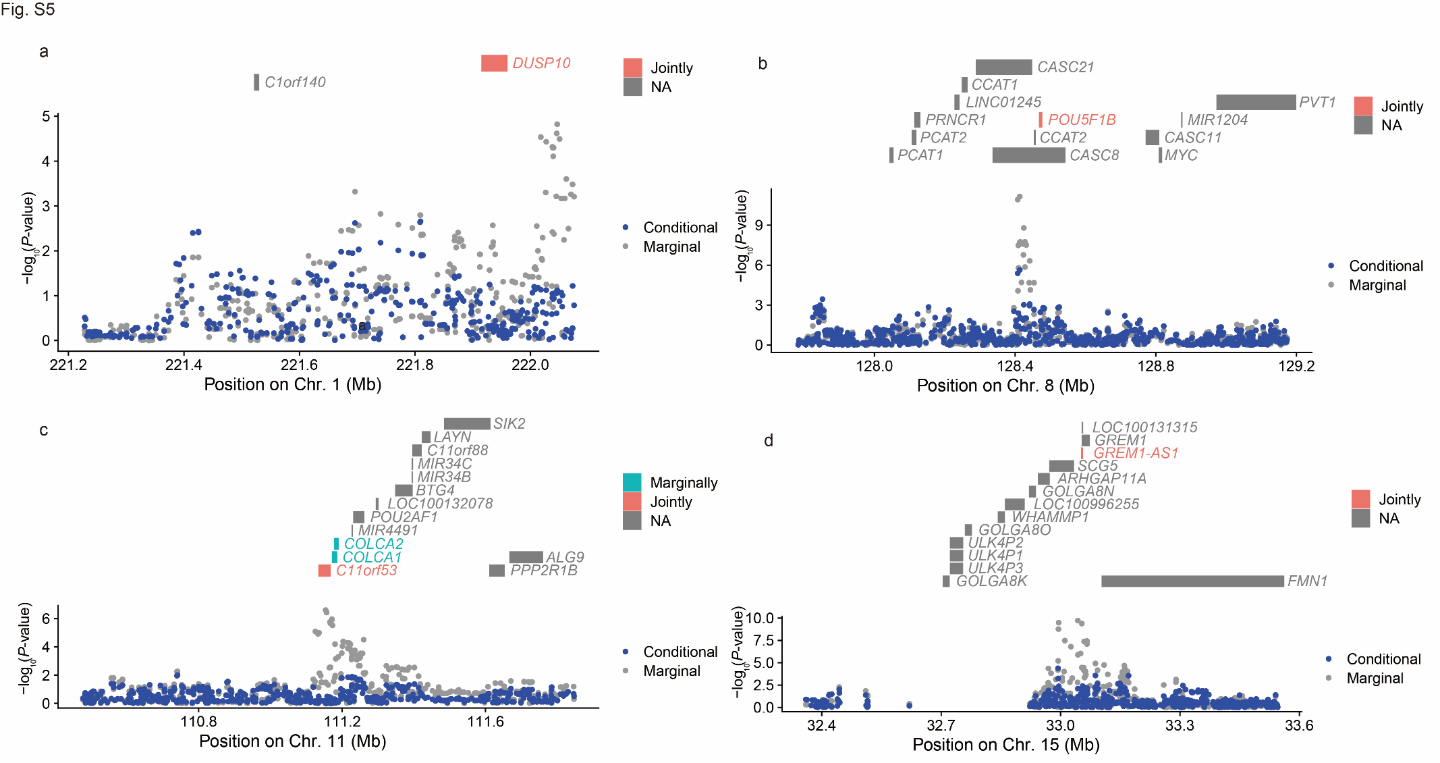


**Fig. S5 Conditional transcriptome-wide association study analysis for loci showing statistically significant associations with multiple genes.** (**a**) Chromosome 1 regional association plot. (**b**) Chromosome 8 regional association plot. (**c**) Chromosome 11 regional association plot. (**d**) Chromosome 15 regional association plot. The top panel shows all of the protein-coding genes or genes in the transcriptome-wide association study. Jointly significant genes are highlighted in red, marginally associated TWAS genes are shown in blue, and genes that were not in the transcriptome-wide association study (TWAS) are in gray. The bottom panel shows a Manhattan plot of the genome-wide association study data before (gray) and after (blue) conditioning on the jointly significant genes. TWAS, transcriptome-wide association study. NA, not available.


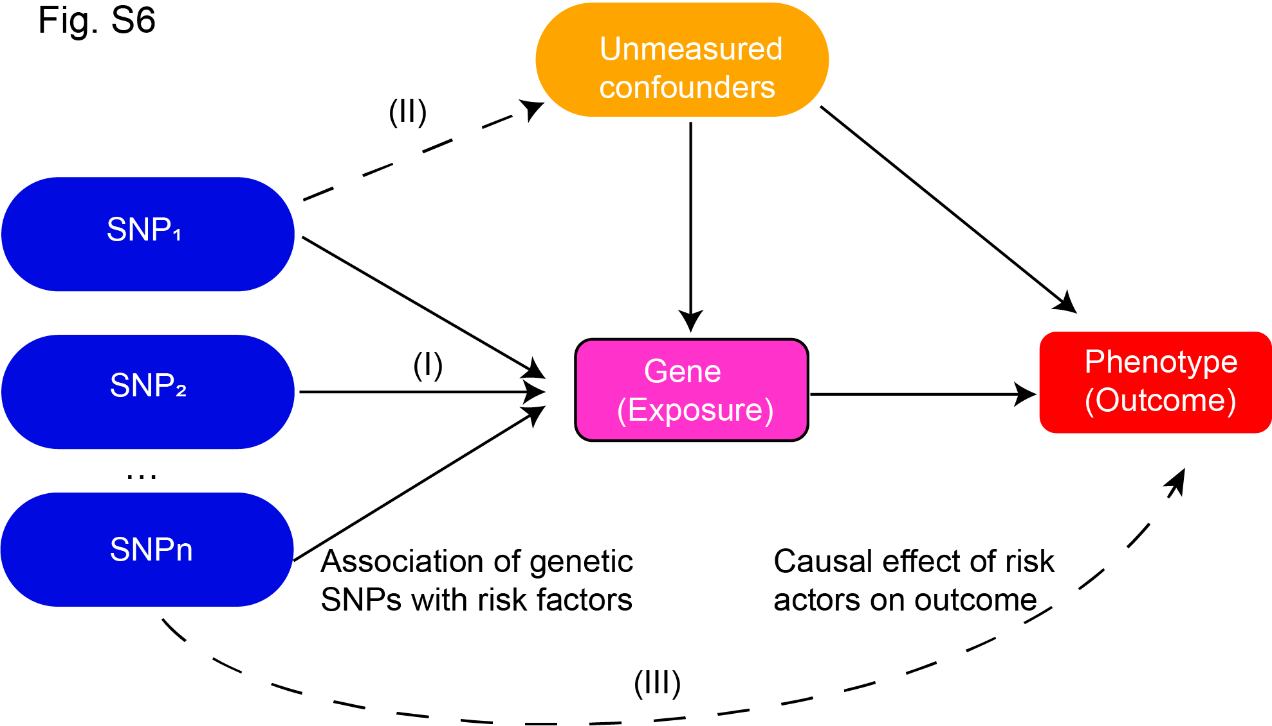


**Fig.S6 Schematic diagram of the** **Summary data-based Mendelian randomization model.** The Summary data-based Mendelian randomization (SMR) software was originally developed to test the pleiotropic association between the expression level of a gene and a complex trait or disease status of interest using summary-level data from GWAS and expression quantitative trait loci (eQTL). In the process of SMR analyses, SNPs were used as instrumental variables, gene expression levels were used as exposure factors, and disease status or traits of the individuals were used as outcome variables.


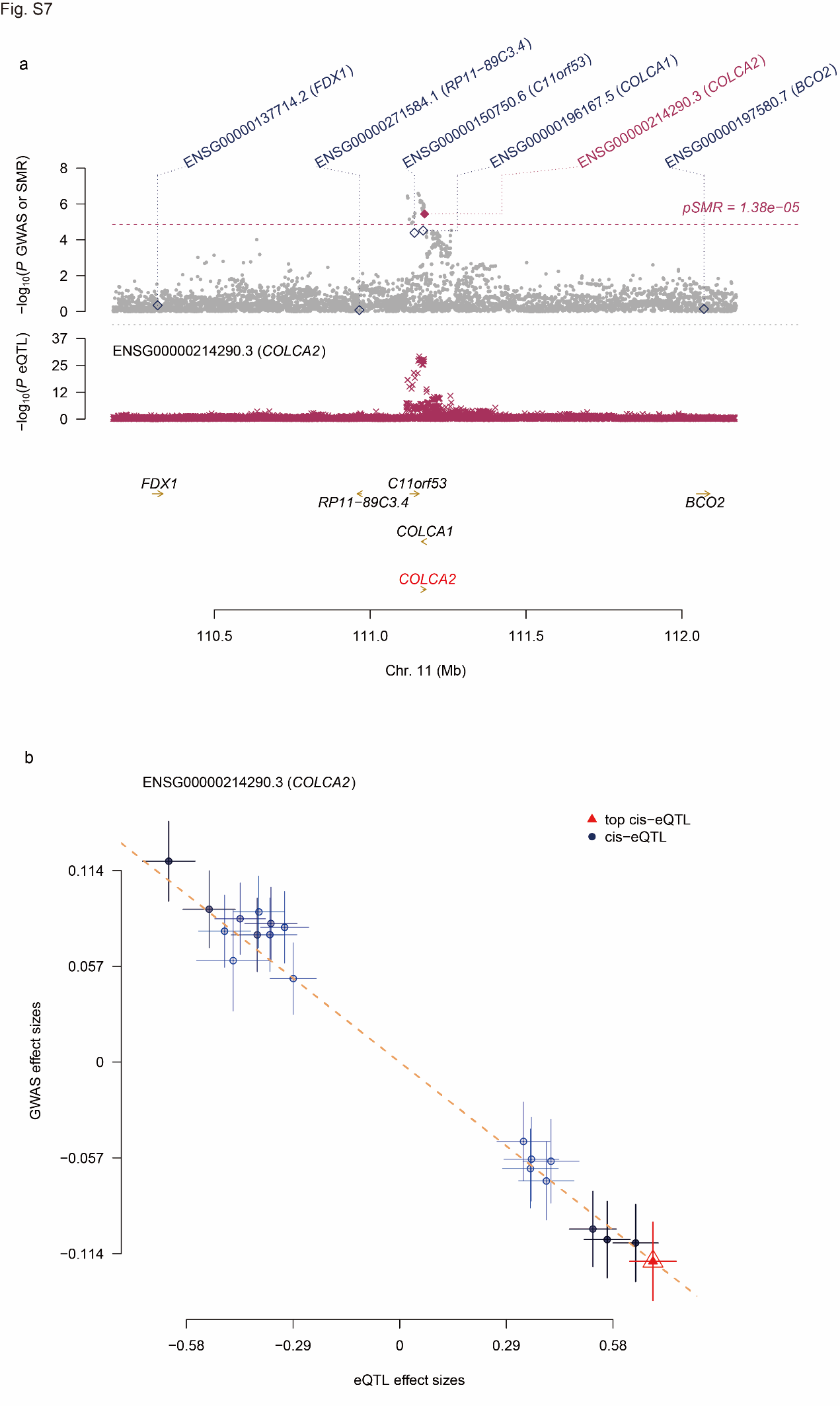


**Fig. S7 Prioritizing genes at the *COLCA2* locus for CRC.** Shown are results from the SMR analysis using the CRC GWAS summary data and the GTEx eQTL summary data in colon transverse tissue. In the top plot, gray dots represent the -log_10_ (*P*) for SNPs from the CRC GWAS summary data, and rhombuses represent the -log_10_ (*P*) for probes from the SMR test with solid rhombuses indicating that the probes pass the HEIDI test and hollow rhombuses indicating that the probes do not pass the HEIDI test. In the middle plot, eQTL results in colon transverse tissue for the probes, tagging *COLCA2*. The bottom plot, location of genes tagged by the probes. Highlighted in maroon indicates probes that pass SMR threshold and lead genes are highlighted in red. CRC, colorectal cancer; eQTL, expression quantitative trait loci; GTEx, Genotype-Tissue Expression Project; GWAS, genome-wide association studies; HEIDI, heterogeneity in dependent instruments; SMR, summary data-based Mendelian randomization.
